# Genetic Analysis of 25 Patients with 5*α*-Reductase Deficiency in Chinese Population

**DOI:** 10.1155/2020/1789514

**Published:** 2020-06-09

**Authors:** Bing Han, Tong Cheng, Hui Zhu, Jie Yu, Wen-jiao Zhu, Huai-dong Song, Haijun Yao, Jie Qiao

**Affiliations:** ^1^Department of Endocrinology, Shanghai Ninth People's Hospital, Shanghai Jiao Tong University School of Medicine, Shanghai, China; ^2^Central Laboratory, Clinical Research Center, Shanghai Ninth People's Hospital, Shanghai Jiao Tong University School of Medicine, Shanghai, China; ^3^Department of Urology, Shanghai Ninth People's Hospital, Shanghai Jiao Tong University School of Medicine, Shanghai, China

## Abstract

**Background:**

A deficiency in steroid 5*α*-reductase type 2 is an autosomal recessive disorder. Affected individuals manifested ambiguous genitalia, which is caused by decreased dihydrotestosterone (DHT) synthesis in the fetus.

**Methods:**

We analyzed 25 patients with 5*α*-reductase deficiency in China. Seventeen of the 25 patients (68%) were initially raised as females. Sixteen patients changed their social gender from female to male after puberty.

**Results:**

Eighteen mutations were identified in these patients. p.Gly203Ser and p.Gln6∗ were found to be the most prevalent mutations. On the basis of the genotype of these patients, we divided them into different groups. There was no significant difference in hormone levels and external masculinization score (EMS) in patients with or without these prevalent mutations. Twelve common single-nucleotide polymorphisms (SNPs) near the p.Gln6∗ mutation were chosen for haplotype analysis. Three haplotypes were observed in 6 patients who had the p.Gln6∗ mutation (12 alleles).

**Conclusion:**

We analyzed mutations of the *SRD5A2* gene in Chinese patients with 5*α*-reductase deficiency. Although hotspot mutations exist, no founder effect of prevalent mutations in the *SRD5A2* gene was detected in the Chinese population.

## 1. Introduction

*SRD5A2*, the gene encoding steroid 5*α*-reductase type 2 (5*α*-RD2), is located on chromosome 2p23 and contains 5 exons [1]. 5*α*-RD2 contains 254 amino acids and is predominantly expressed in fetal genital skin, male productive tissues, and the prostate [2]. It converts testosterone (T) to dihydrotestosterone (DHT), which plays a vital role in the development of male external genitalia [3].

5*α*-RD2 deficiency is an autosomal recessive disorder caused by mutations in the *SRD5A2* gene that impair the conversion of T to DHT. Since first described in 1974 [4, 5], more than 100 mutations of *SRD5A2* have been identified worldwide [6–8]. Phenotypes of 5*α*-RD2 deficiency range from female external genitalia to hypospadias with microphallus to apparently normal male external genitalia. After puberty, these patients showed different degrees of virilization and often change their social gender from female to male [9].

In some cases, 5*α*-RD2 deficiency shared the same clinical phenotype with androgen insensitivity syndrome, which may have caused misdiagnosis of this disease. Elevated baseline and hCG-stimulated T/DHT ratio is useful to diagnose this disease [10–12]. However, more accurate diagnoses rely on genetic testing, especially in prepubertal patients [13].

In this study, we analyzed 25 Chinese patients with 5*α*-RD2 deficiency. Eighteen mutations in the *SRD5A2* gene were identified from these patients. In these mutations, p.Gln6∗ was widely reported. Therefore, we chose 12 SNPs near p.Gln6∗ and performed haplotype analysis to determine whether this mutation has a founder effect in the Chinese population.

## 2. Patients and Methods

### 2.1. Patients

Twenty-five patients, aged from 4 days to 34 years (diagnosis age), with 46,XY DSD were included in this study. Patients 1-14 were reported by Cheng et al. [14]. Patients 15-25 were reported by Zhu et al. [9]. Patients 4 and 17 came from consanguineous families. Patients 8 and 9 were twin brothers with similar phenotypes, whereas patients 21 and 22 were siblings with different phenotypes. The clinical and definitive diagnosis of steroid 5*α*-reductase-2 deficiency was described previously [9]. External masculinization score (EMS) was also accessed as previously described [15]. This study was approved by the Ethics Committee of our institute. Written informed consent was obtained from all the adult patients themselves or from the parents of patients who were children.

### 2.2. Mutation Analysis

Genomic DNA of the patients was extracted from peripheral blood leukocytes using a kit (TIANGEN Biotech, Beijing, China). All five exons of the *SRD5A2* gene were amplified as previously described [9]. Then, the PCR products were purified by shrimp alkaline phosphatase (SAP, USB, Cleveland, USA) and directly sequenced in both directions. When a novel mutation was found, PCR fragments amplified from the genomic DNA of 100 healthy subjects were also analyzed to exclude polymorphism.

### 2.3. Haplotype Analysis

For haplotyping analysis, 12 common single-nucleotide polymorphisms (SNPs) near the location of p.Gln6∗ were selected from the *SRD5A2* gene based on the genotyping data from 1468 Chinese Han individuals in a genome-wide association study (GWAS) [16] (rs12470143: SRD5A2 RefSeq Gene NG_008365.1:g.47484G>A; rs12470196: SRD5A2 RefSeq Gene NG_008365.1:g.47290G>A; rs57971483: SRD5A2 RefSeq Gene NG_008365.1:g.45532A>G; rs4952220: SRD5A2 RefSeq Gene NG_008365.1:g.45486G>A; rs2300697: SRD5A2 RefSeq Gene NG_008365.1:g.24405G>A; rs2300698: SRD5A2 RefSeq Gene NG_008365.1:g.24249T>C; rs2300699: SRD5A2 RefSeq Gene NG_008365.1:g.24075C>A; rs2300700: SRD5A2 RefSeq Gene NG_008365.1:g.24050C>T; rs2300701: SRD5A2 RefSeq Gene NG_008365.1:g.24034T>C; rs522638: SRD5A2 RefSeq Gene NG_008365.1:g.5367T>C; rs523349: SRD5A2 RefSeq Gene NG_008365.1:g.5336C>G; rs632148: SRD5A2 RefSeq Gene NG_008365.1:g.5010G>C) (http://www.ncbi.nlm.nih.gov/SNP/). Two twin brothers (patients 8 and 9) had the same genotype. Therefore, six patients carrying the p.Gln6∗ heterozygous mutation were genotyped for these 12 SNPs by PCR and Sanger sequencing. PHASE 2.1 software was used to perform the haplotyping analysis. We also compared the difference in haplotypes between healthy controls and our patients.

### 2.4. Statistical Analysis

Data were expressed as the means ± SD. Statistical analysis was determined using the unpaired Student's *t*-test. The analyses were performed using SPSS version 11.0. *P* < 0.05 was considered significant.

## 3. Results

### 3.1. Clinical Characteristics

The molecular analysis results of 25 patients with 5*α*-RD2 deficiency are summarized in Table 1. The detailed clinical characteristics were described previously [9, 14]. Seventeen of the 25 patients (68%) were initially raised as females. Among them, 16 patients (94.1%) had changed their social gender from female to male. Six patients were prepubertal (patients 10, 11, 15, 16, 17, and 18), and only one (patient 15) was raised as a male before the first examination. Nineteen patients were diagnosed at the age of more than 16 years old. Patients 21 and 22 were siblings but presented with different phenotypes. Isolated micropenis or normal female genitalia were found in patients 16 and 22. The topical application of DHT gel was used by patients 3, 4, 5, 10, 16, and 20 with different diagnostic ages. Six patients were from Guizhou province and four patients were from Jiangxi province. Of the seven patients with p.Gln6∗ mutation, three were from Guizhou province and the other four patients were from Sichuan, Anhui, Hubei, and Shandong provinces.

### 3.2. DNA Sequencing

Eighteen different mutations were identified in 25 patients from 23 unrelated families (Table 1). Compound heterozygous mutations were found in 15 patients, and homozygous mutations were found in 7 patients. The most frequent mutations in our study was p.Gly203Ser and p.Gln6∗, found on one allele of 8 (patients 2, 4, 10, 16, 20, 21, 22, and 25) and 7 patients (patients 1, 6, 8, 9, 11, 12, and 25). The reported polymorphism, p.Val89Leu, was identified in 14 patients.

### 3.3. Comparison of Hormone Levels and EMS between Different Mutation Types

To investigate the influence of “hotspot” mutations p.Gly203Ser and p.Gln6∗ on hormone levels, we compared patients with these two mutations and those without the two mutations. In a comparison of 8 patients carrying the p.Gly203Ser mutation and 17 patients carrying other mutations, LH, FSH, T, DHT, T/DHT, and EMS showed no significant difference (*P* ≥ 0.05) in Table 2. We also compared 7 patients carrying the p.Gln6∗ mutation and 18 patients without the p.Gln6∗ mutation. The results indicated that LH, FSH, T, DHT, and T/DHT were not significantly different (*P* ≥ 0.05). However, EMS showed marginal difference between two groups (*P* = 0.05) (Table 3).

### 3.4. Haplotype Analysis

The heterozygous nonsense mutation p.Gln6∗, which was the most frequent mutation in this study, was identified in 7 cases from 6 pedigrees (7/25, 28%). To identify potential common ancestors for those subjects who carried this mutation, twelve SNPs in and around exon 1 of *SRD5A2* spanning 26 kb were chosen for analysis. Three haplotypes were observed in 6 patients (12 alleles), with CCTCCAGGAAGC and TTCATGTAGGCG in 5 alleles, respectively, whereas CCTCTGTGGGCG were found in 2 alleles. It cannot be concluded that the p.Gln6∗ mutation has a founder effect in the Chinese population since in 6 alleles carrying the p.Gln6∗ mutation, two different haplotypes were found (haplotype 1 and haplotype 3), and haplotype 1 was also found to be common in 1494 normal alleles (Table 4).

## 4. Discussion

DHT, converted from testosterone in the skin of the fetal labioscrotal folds as a paracrine factor, induces labioscrotal fusion, the development of the phallic urethra, and phallic enlargement [17]. Two 5*α*-reductases in humans, encoded by different genes on chromosome 5 and chromosome 2 (*SRD5A1* and *SRD5A2*), were identified to catalyze the conversion of testosterone to DHT. Both enzymes are hydrophobic and membrane bound [1] and have proven difficult to solubilize in active form. With a similar gene structure of 5 exons separated by 4 introns, *SRD5A2* was found to be predominantly expressed in the male urogenital tract and prostate and in female genital skin, whereas *SRD5A1* is predominantly expressed in the ovary, testis, liver, and nongenital skin. It is well known that dehydroepiandrosterone (DHEA) and androstenedione or androstenediol are key intermediates in the synthesis of testosterone, and fetal DHT is produced in situ in its target organ in the “classic” pathway of androgen synthesis. Recently, an alternative, or “backdoor,” biosynthetic pathway was reported to exist, leading to the production of DHT in the fetal testis, in which progesterone was converted to 5*α*-dihydroprogesterone by SRD5A1 and 3*α*-hydroxysteroid dehydrogenase (aldo-keto reductase (AKR)) [18, 19]. The finding that human *AKR1C2* mutations can result in 46,XY DSD shows that DHT produced in the fetal testis via the backdoor pathway acts as a hormone that collaborates with DHT produced in the genital skin by *SRD5A2* to induce labioscrotal fusion [19].

In patients 1 and 2, they all have three mutations. In the first patient, a cloning experiment indicated that p.Gln6∗and p.Lys35Asn were located in the same chromosome. Due to the severe truncating mutation at codon 6 (p.Gln6∗), the latter mutation (p.Lys35Asn) could not be expressed in the mature protein. So, p.Gln6∗ and p.Phe234Leu were pathogenic mutations in this patient. In the second patient, p.Gly203Ser and p.Arg227Gln were located in different chromosomes. We cannot perform a cloning test because of the long distance between p.Gly34Arg and p.Gly203Ser/p.Arg227Gln on the chromosome. In a previous study, functional analysis showed that p.Gly34Arg only retained about 1.2% of the enzyme activity [20]. So, all three mutations caused inactivity of the enzyme in the second patient.

To be noted, 17 (68%) out of 25 patients were raised initially as females in our study. Of these 17 patients, 16 (94.1%) changed their social gender to male. However, the ratios of female social sex and female-to-male sex change range from 77 to 93% and 12 to 50%, respectively, in Brazilian and French studies [21]. In a Chinese study, Song et al. analyzed 86 children (age 2 months to 17 years) with 5*α*-reductase deficiency and found that 27 (31.4%) out of the 86 patients were raised as females [22]. Cheng et al. found 4 (8.9%) out of 45 Chinese patients (age 3 months to 12 years) were raised as females [23]. These two Chinese studies did not refer to the female-to-male social sex change. The female social sex ratio of our patients was similar to that in a French study [24] but was higher than those in two Chinese studies. However, the ratio of female-to-male sex change in our study was obviously higher than those in Brazilian and French studies. This might be attributed to the age of diagnosis [21]. In the Brazilian and French studies, the patients were diagnosed at an average age of 16 and 7.6 years old, respectively. Thus, the prevalence of social sex alteration in the Brazilian study was higher than that in the French study (50% vs. 12%). In our study, 19 patients (76%) were older than 16 years and the average age at diagnosis was 18 years. So the ratio of social sex change was higher than the Brazilian study. Besides that, most of our patients were from remote areas (such as Guizhou and Jiangxi) and presented with female external genitalia. Poor medical conditions and insufficient attention of parents might also be considered.

In our study, p.Gly203Ser and p.Gln6∗ were the most common mutations. p.Gly203Ser was identified in 8 patients with an allele frequency 20.8% and was homozygous in nonconsanguineous patients 4 and 10. Zhang et al. [25] identified the heterozygous mutation p.Gly203Ser in 3 patients and presumably suggested a founder effect in the Chinese population. However, in our previous study [9], we found that p.Gly203Ser in *SRD5A2* was the most prevalent mutation and that there was no founder effect of this mutation in the Chinese population. In this study, seven patients from 6 unrelated families were heterozygous for the nonsense mutation p.Gln6∗, which was reported mainly in East Asia.

For haplotype analysis, we selected 12 SNPs near p.Gln6∗ based on the genotyping data from 1468 Chinese Han individuals in GWAS data [16]. Actually, these 12 SNPs could form more than 3 haplotypes, but we only listed 3 haplotypes which were found in 6 patients with p.Gln6∗ mutation in Table 4. Mutant allele T (p.Gln6∗) could be found both in haplotypes 1 (5 alleles) and 3 (1 alleles). Moreover, wild genotype C and mutant genotype T simultaneously presented in haplotype 3. So, there was no linkage disequilibrium between p.Gln6∗ and the other 12 SNPs. Besides, no geographic relationship was found in 5 patients who shared haplotype 1. Therefore, p.Gln6∗ does not appear to derive from a common ancestor in the Chinese population, which is consistent with the fact that it has been reported in Mexico, China, Korea, and Thailand [8, 9, 13, 26]. The recurrence of a particular mutation in a geographically isolated region suggests a founder effect; for example, two specific *CYP17A1* mutations in China or East Asia have been rarely reported in other ethnic groups [27, 28]. Similarly, two population-specific mutations in *CYP17A1* (p.Trp406Arg and p.Arg362Cys) also showed a founder effect in Brazil [29].

The p.Arg246Gln substitution that we found in patients 3, 17, 18, and 19 seems to be a hot spot of *SRD5A2* mutations since it has been reported in patients with diverse geographic and ethnic backgrounds, including those with Korean, Indian, Mexican, and Italian ethnic backgrounds [13, 30–32]. Eunice et al. [33] even proposed a founder gene effect of this mutation in India. Functional studies of this mutation suggested that it decreases the affinity for the cofactor NADPH [1, 34]. The optimal pH of the mutant enzyme is also changed, resulting in a disruption of the activity of the enzyme. Missense mutations that result in less than 0.4% activity of the 5*α*-reductases usually result in ambiguous or female external genitalia [26]. p.Arg227Gln was also detected in four patients (patients 2, 10, 13, and 15). Cheng et al. [23] reported that the p.Arg227Gln mutation was most frequently reported (91.1%) in South China, which suggested a founder effect. However, the incidence of p.Arg227Gln was relatively lower in our cohort (4/25).

There were some less frequent mutations detected in our study, such as p.Gly34Arg and c.182-2A>G. p.Gly34Arg was identified only in one patient (patient 2). However, this mutation was prevalent in Egypt and had linkage disequilibrium with the V89L polymorphism, which indicated a founder effect of the p.Gly34Arg mutation among Egyptians [35]. A c.182-2 A>G mutation was detected in a set of siblings (patients 21 and 22); this mutation is common in Greek-Cypriot patients with 5*α*-reductase deficiency and is very likely to be the result of a founder effect [36, 37]. Vilchis et al. [38] analyzed 11 Mexican patients with steroid 5*α*-reductase 2 deficiency. They found that 40% of the mutant alleles (9/22) contained the gene variant p.Pro212Arg and hypothesized that the presence of this mutation may constitute a founder gene effect. However, we did not discover this mutation in our cohort.

## 5. Conclusion

Eighteen mutations were identified in 25 Chinese patients with 5*α*-RD2 deficiency. p.Gln6∗ and p.Gly203Ser were the most prevalent mutations. Although no homozygous p.Gln6∗ mutation and only two homozygous p.Gly203Ser mutations were included, the patients with or without p.Gln6∗ and p.Gly203Ser did not demonstrate a significant difference in hormone levels. Finally, no founder effect of the p.Gln6∗ mutation in the *SRD5A2* gene was detected in the Chinese population.

## Figures and Tables

**Table 1:** Clinical and genetic characteristics of the patients.

Patient no.	Location	Age of diagnose	Sex of rearing at birth	EMS	*SRD5A2* mutation
1	Sichuan	22 yr	F to M	3	p.Gln6∗/p.Lys35Asn/p.Phe234Leu
2	Guizhou	23 yr	M	6	p.Gly203Ser/p.Arg227Gln/p.Gly34Arg
3	Sichuan	20 yr	M	5	p.Leu20Pro/p.Arg246Gln
4	Jiangxi	19 yr	M	6	p.Gly203Ser/p.Gly203Ser
5	Liaoning	23 yr	F to M	2	p.Ala228Val/---
6	Guizhou	24 yr	F	2	p.Gln6∗/---
7	—	30 yr	M	2	p.Tyr136∗/p.Tyr136∗
8	Guizhou	18 yr	F to M	2	p.Gln6∗/p.His162Pro
9	Guizhou	18 yr	F to M	2	p.Gln6∗/p.His162Pro
10	Guizhou	5 yr	F to M	2	p.Gly203Ser/p.Gly203Ser
11	Anhui	11 yr	F to M	2	p.Gln6∗/p.Arg227Gln
12	Hubei	23 yr	F to M	2	p.Gln6∗/p.Asn193Ser
13	Yunnan	18 yr	M	5	p.Arg171Ser/p.Gly196Val
14	Guizhou	34 yr	F to M	3	p.Leu20Pro/p.Arg227∗
15	Jiangxi	4 yr	M	9	p.Val89Leu/c.655del
16	Jiangsu	4 d	F to M	3	p.Gly203Ser/p.Arg227Gln
17	Henna	14 yr	F to M	2.5	c.698+2T>C/c.698+2T>C
18	Zhejiang	12 yr	F to M	2.5	p.Arg246Gln/p.Arg246Gln
19	Anhui	18 yr	F to M	2.5	p.Arg246Gln/p.Asn193Ser
20	Jiangxi	17 yr	F to M	3	p.Gly203Ser/p.Arg246Gln
21	—	18 yr	M	9	p.Gly203Ser/c.182-2A>G
22	—	23 yr	F to M	3	p.Gly203Ser/c.182-2A>G
23	Jiangxi	16 yr	F to M	3	p.Ala228Val/p.Ala228Val
24	Henan	23 yr	F to M	2.5	p.Ala228Val/p.Ala228Val
25	Shandong	16 yr	F to M	2	p.Gln6∗/p.Gly203Ser

F: female; M: male; EMS: external masculinization score. --- indicates no mutation in another chromosome.

**Table 2:** Comparison of hormone levels between patients with and without p.GLY203SER mutation.

	GLY203SER (*n* = 8)	Other variants (*n* = 17)	*P* value
LH	6.58 ± 4.24	8.82 ± 6.00	0.36
FSH	5.94 ± 3.03	14.36 ± 11.09	0.05
T	19.27 ± 12.97	20.74 ± 11.25	0.77
DHT	0.56 ± 0.37	0.49 ± 0.28	0.61
T/DHT	40.28 ± 26.10	52.05 ± 34.17	0.41
EMS	4.25 ± 2.49	3.06 ± 1.80	0.19

EMS: external masculinization score.

**Table 3:** Comparison of hormone levels between patients with and without p.GLN6∗ mutation.

	GLN6∗ (*n* = 7)	Other variants (*n* = 18)	*P* value
LH	8.47 ± 6.53	7.91 ± 5.21	0.83
FSH	16.35 ± 12.47	9.85 ± 8.65	0.15
T	20.25 ± 12.29	20.28 ± 11.65	1.00
DHT	0.35 ± 0.19	0.57 ± 0.33	0.15
T/DHT	64.03 ± 41.28	42.28 ± 26.49	0.15
EMS	2.14 ± 0.38	3.94 ± 2.25	0.05

EMS: external masculinization score.

**Table 4:** Frequencies of the haplotypes with p.Gln6∗ mutation in 12 cases and 1468 controls.

SNP	Mutation	Location	Chr. position	Nucleotide change	Haplotype 1	Haplotype 2	Haplotype 3	
Rs12470143		Intron 1	31763558	C>T	C	T	C	
Rs12470196		Intron 1	31763752	C>T	C	T	C	
Rs57971483		Intron 1	31765510	C>T	T	C	T	
Rs4952220		Intron 1	31765556	A>C	C	A	C	
Rs2300697		Intron 1	31786637	C>T	C	T	T	
Rs2300698		Intron 1	31786793	A>G	A	G	G	
Rs2300699		Intron 1	31786967	G>T	G	T	T	
Rs2300700		Intron 1	31786992	A>G	G	A	G	
Rs2300701		Intron 1	31787008	A>G	A	G	G	
Rs522638		Intron 1	31805675	A>G	A	G	G	
Rs523349		Exon 1	31805706	C>G	G	C	C	
Rs632148		5′-UTR	31806031	C>G	C	G	G	
Control (allele)					1494	250	936	
	p.Gln6∗	Exon 1	31805954	C>T	T	C	C	T
Case (allele)					5	5	1	1

## Data Availability

Data are available upon request to corresponding author.

## Abbreviations

SNPs: Single-nucleotide polymorphisms
5*α*-RD2: 5*α*-Reductase type 2
DHT: Dihydrotestosterone
GWAS: Genome-wide association study.
